# Design of Progressive Addition Lens for Presbyopia: A Systematic Review

**DOI:** 10.1007/s44402-026-00056-w

**Published:** 2026-04-22

**Authors:** Jijing Li, Yue Wu, Mingguang He

**Affiliations:** 1 Centre for Eye and Vision Research (CEVR), Shatin, Hong Kong China; 2 School of Optometry, The Hong Kong Polytechnic University, Kowloon, Hong Kong China; 3 Research Centre for SHARP Vision (RCSV), The Hong Kong Polytechnic University, Kowloon, Hong Kong China

**Keywords:** Presbyopia, Progressive addition lenses (PALs), Systematic review

## Abstract

**Purpose:**

Presbyopia is a common age-related condition and progressive addition lenses (PALs) are a primary corrective method. This systematic review synthesised recent evidence (2015–2025) on the technical design of PALs for presbyopia, focusing on key developments in design methodologies, performance evaluation and emerging lens concepts.

**Methods:**

A systematic search of six databases (PubMed, Embase, Web of Science, Cochrane Library, Scopus and SPIE Digital Library) was conducted following PRISMA guidelines. Eligible studies addressed PALs design principles, algorithms, methodologies or optical performance evaluation. Study quality was assessed using a customised 12-dimensional framework.

**Results:**

The review included 17 eligible studies. Four major thematic areas of advancement were identified: the maturation of freeform surface design as the primary method for creating complex optical surfaces; the evolution of strategies for the control of surface astigmatism; the development of advanced optical performance quantification techniques enabling objective quantification of lens performance and the emergence of novel lens concepts expanding design possibilities beyond conventional refractive approaches. Key progress was noted in algorithmic optimisation for the control of surface astigmatism and the shift from subjective assessment to data-driven performance validation.

**Conclusion:**

PAL design has evolved significantly toward customised, algorithm-driven solutions. However, challenges remain in standardising clinical fitting, understanding neural adaptation and developing personalised designs for diverse presbyopic subpopulations. Future progress will hinge on deeper interdisciplinary integration and ongoing technological innovation to evolve PALs from standardised corrections into adaptive visual wearables.

Key Points

- This review highlights how advanced lens design methods now allow for more personalised and comfortable vision correction for people with presbyopia.
- New ways to measure lens performance are making it possible to match lens designs better to real-world visual needs, but user comfort still depends on individual adaptation.
- Future progress in vision correction will require combining technology, clinical practice and an understanding of how the brain adapts, to create truly personalised solutions.

## Introduction

Presbyopia is a common age-related condition characterised by a progressive decline in the eye’s ability to focus on nearby objects due to loss of lens accommodation [1]. As a natural part of aging, presbyopia affects virtually everyone from their 40s onwards. The global population experiencing presbyopia has been growing significantly, affecting between 1.09 billion and 1.80 billion people [2–4]. This condition primarily impacts individuals whose occupations or daily activities require near vision, such as reading, using digital devices and fine manual work [5]. As a result, a range of corrective interventions, including reading glasses, contact lenses and surgical options, have been developed to manage this widespread issue [6].

Presbyopic spectacles represent the primary corrective method for presbyopia due to their non-invasiveness and convenience [7]. Traditional designs, such as bifocals and trifocals, are types of multifocal lenses that correct vision for both distance and near, incorporating distinct optical zones with visible lines on the lenses [7]. However, these visible lines are not only cosmetically unappealing but also cause an “image jump” phenomenon. This occurs when the wearer’s gaze crosses between the different power zones, leading to a sudden shift in the perceived position of objects, visual discontinuity and general discomfort [8]. To overcome these limitations, progressive addition lenses (PALs), also known as progressive lenses, represent an advanced form of multifocal lenses [9]. By providing a seamless, gradual transition of optical power across various distances, PALs significantly enhance visual comfort and quality of life for wearers [10].

Despite providing a superior visual correction experience, PALs still present significant design challenges. These lenses incorporate a continuous gradient of optical power across a curved surface, with defined refractive power and astigmatism at every point through the distance, intermediate and near zones [11]. However, this smooth power progression inevitably introduces peripheral aberrations, which can cause blurred peripheral vision [12] or a “swimming sensation”, a phenomenon where objects appear distorted or seem to move or float, particularly during the wearer’s initial adaptation period [13].

Consequently, optimising PAL designs to minimise these peripheral aberrations, widen the clear field of vision and enhance wearing comfort remains a key focus in optical research. Many current studies are dedicated to balancing the continuous distribution of refractive power and astigmatism with effective control over peripheral aberrations [11]. This systematic review aims to review PAL designs comprehensively by analysing recent advancements in freeform surface design, control of surface astigmatism, optical performance quantification and novel concepts, as well as highlight key trends and identify existing gaps. Furthermore, this review will critically assess the quality of the included literature to provide a comprehensive overview of the state of the field.

## Methods

This systematic review was conducted in accordance with the Preferred Reporting Items for Systematic Reviews and Meta-Analyses (PRISMA) guidelines. The study protocol was prospectively registered in the International Prospective Register of Systematic Reviews (PROSPERO) under the ID CRD420251130801.

This systematic review was designed to identify and synthesise research related to the design of PALs for presbyopia correction. Eligible studies were required to address the principles, algorithms, methodologies or strategies for PALs design or technologies for evaluating their optical performance and visual perception. Only original research articles were considered, including theoretical modelling, computational simulations, laboratory experiments, methodological developments and engineering applications. Studies had to report qualitative or quantitative outcomes relevant to the lens design.

A comprehensive literature retrieval was conducted across six databases: PubMed, Embase, Web of Science, Cochrane Library, Scopus and SPIE Digital Library. To incorporate the most recent and relevant studies, the search was restricted to publications from January 2015 to August 2025. A set of keywords related to presbyopia and PALs was combined for the search, with a primary focus on English-language articles. Additionally, forward citation tracking was performed by reviewing all documents that cited the studies initially included. A detailed account of the search strategy is available in the Supplementary Materials.

All citations identified via electronic database searches were imported into EndNote (endnote.com) by one author (J.L.), after which duplicate entries were eliminated. Two reviewers (J.L. and Y.W.) independently screened titles and abstracts to evaluate study eligibility and then performed a full-text review of potentially relevant articles to confirm final inclusion. Data extraction was conducted using a standardised form to collect key information, including authors, publication year, country or region of origin, study type and PAL design or optical evaluation. The initial data extraction was completed by one author (J.L.), with independent verification by a second author (Y.W.). Any discrepancies between the two reviewers were resolved through discussion or by consulting a third reviewer (M.H.) if necessary.

Given the absence of a universally recognised quality assessment tool specifically tailored to the diverse types of studies included in this systematic review, a customised evaluation framework comprising 12 distinct dimensions was established (see Supplementary Material). This framework primarily addressed two key aspects: the rigour and reliability of the research, as well as the value and impact of the research findings. Two reviewers (J.L. and Y.W.) independently assessed each study. Studies rated as “high quality” demonstrated excellence, sufficiency or transparency across the criteria; those rated as “medium quality” showed acceptable performance but with notable limitations and studies classified as “low quality” contained serious flaws or critical information gaps. Any discrepancies between the reviewers were resolved through discussion or by consulting a third reviewer (M.H.).

## Results

### Study Selection and Characteristics

**Fig. 1:**
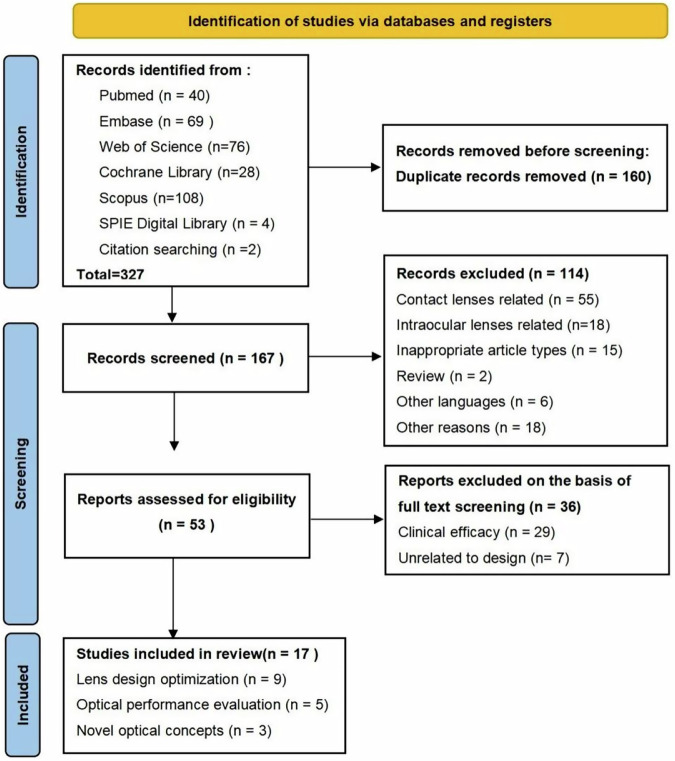
Preferred Reporting Items for Systematic Reviews and Meta-Analyses (PRISMA) flow diagram of the study selection process.

A total of 327 articles were initially retrieved via database searches and citation tracking. After excluding 160 duplicates, 167 articles proceeded to preliminary screening based on titles and abstracts. Among these, 114 articles were excluded for reasons including irrelevant topics (e.g., exclusive focus on contact lenses or intraocular lenses), inappropriate article types (e.g., meeting abstracts or reviews) or language restrictions. Subsequently, the full texts of the remaining 53 articles were evaluated. During this stage, 29 articles were excluded for solely investigating the clinical efficacy of general PALs, while another seven were removed for lack of relevance to the PAL design. Ultimately, 17 articles were included in this systematic review. The flowchart of the selection process is shown in Fig. 1.

The characteristics of the 17 included studies were summarised in Table 1. These studies were categorised into four thematic areas based on their primary contribution to PAL design. Four studies addressed freeform surface design, which was central to modern PALs as it provided the methodological foundation for creating complex, customised optical surfaces that aimed to deliver a continuous and natural visual experience across distances. Five studies focused on the control of surface astigmatism, which was a fundamental design challenge. The strategies developed in this area directly targeted the reduction of peripheral blur and distortion, which were critical for enhancing wearer comfort and clinical acceptance. Another five studies contributed to optical performance quantification, facilitating a shift towards data-driven design by establishing objective metrics and tools to evaluate lens performance. Finally, three studies explored emerging lens concepts, investigating frontier technologies that proposed innovative pathways to overcome limitations of conventional designs, such as lens thickness and restricted depth-of-focus.

**Table 1:** Characteristics of eligible studies.

Authors	Year	Country/Region	Study type	Lens design/Optical evaluation
Xiang et al. [15]	2015	China	Proof of concept	Multi-optical-axis freeform design with the intermediate zone filled with small pupils to form the progressive corridor
Casanellas and Castro [16]	2020	Spain	Methodology optimisation	Progressive addition lenses (PALs) optimisation model using spherical coordinates and interior-point solvers
Galinier et al. [17]	2024	France	Frontier conceptual design	Freeform surface generation based on a Fermat’s spiral focal length distribution combined with a quadratic phase function
Yin et al. [18]	2025	China	Methodology optimisation	Customised freeform PAL design method based on localised precision optimisation
Wei et al. [14]	2015	China	Proof of concept	Cylindrical surface superposition for astigmatism correction
Qiu and Cui [19]	2015	China	Proof of concept	Hyperbolic tangential power distribution with Laplace-based astigmatism control
Xia et al. [20]	2022	China	Practical innovation	Curvature-guided segmented freeform surface (SFS) method for correcting astigmatism in blending zones.
Xiang et al. [21]	2022	China	Systematic comparative study	Eighth-order polynomial curvature law for power distribution
Pan et al. [22]	2025	China	Methodology optimisation	Non-Uniform Rational B-Spline (NURBS) -based error functional optimisation for astigmatism reduction
Xiang et al. [23]	2018	China	Methodology optimisation	Multi-optical-axis measurement using Hartmann–Shack wavefront sensor
Perucho et al. [24]	2015	Spain	Practical innovation	Bilayer permanent engraved marks (PEMs) inspection via manual wavefront holoscopy
Perucho et al. [25]	2016	Spain	Practical innovation	Gabor holography-based marking reader for PEMs identification
Ferrer-Altabás et al. [26]	2022	Spain	Practical innovation	Gabor holography adaptation to manual focimeter (Shadowfocimetry) for mark visualisation
De Lestrange-Anginieur et al. [11]	2021	Hong Kong	Methodology optimisation	Moiré deflectometry and vector analysis for astigmatic PAL evaluation
Trapp et al. [29]	2018	Germany	Frontier conceptual design	Dual- Holographic Optical Element (HOE) configuration with dispersion compensation and angular bandwidth enhancement
Trapp et al. [28]	2018	Germany	Frontier conceptual design	Fourier Modal Method (FMM) - based HOE design with grating-parameter-controlled spherical power
Lyu et al. [30]	2024	South Korea	Frontier conceptual design	Periodic power profile-based extended depth-of-focus (EDoF) lens design

Figure 1 outlines the identification, screening, eligibility and inclusion phases. Of the 327 records identified, 17 studies were ultimately included in the systematic review.

### Quality Assessment of Eligible Studies

The quality assessment results (Table 2) indicated that most studies included were of high overall quality. They consistently demonstrated strengths in theoretical rigour, measurement validity and comprehensive performance evaluation, reflecting a mature foundation in core optical modelling and quantitative analysis within PAL design research. However, common limitations were identified. Research process transparency and reproducibility of results were frequently rated as ‘Moderate’ or ‘Low,’ as seen in early proof-of-concept studies [14, 15] where methodological descriptions were brief. Furthermore, dimensions such as control of confounding factors and discussion of limitations or biases were often addressed inadequately. While many studies demonstrated high innovation and originality as well as technical feasibility, explicit consideration of potential biases, such as discrepancies between simulated and real-world conditions, was frequently lacking.

**Table 2:** Quality assessment of eligible studies.

Assessment criterion	Research rigour & credibility						Validity & impact of findings						Overall assessment
	Sample size/Model parameter rationality	Validity and reliability of measurement tools	Appropriateness of data analysis methods	Control of confounding factors	Research process transparency	Reproducibility of results	Comprehensiveness of performance evaluation	Comparative analysis with existing techniques	Innovation and originality	Technical feasibility and implementability	Theoretical rigour and depth	Discussion of limitations or biases	
Xiang et al. [15]	Moderate	High	Moderate	Low	Low	Moderate	Moderate	Moderate	Moderate	High	Moderate	Moderate	Moderate
Casanellas and Castro [16]	High	High	High	Moderate	Moderate	Moderate	High	High	High	High	High	High	High
Galinier et al. [17]	High	High	High	Moderate	Moderate	High	High	High	High	High	High	High	High
Yin et al. [18]	High	High	High	Moderate	Moderate	Moderate	High	High	High	High	High	Moderate	High
Wei et al. [14]	Moderate	Moderate	Moderate	Low	Low	Low	Moderate	Low	Moderate	Moderate	Moderate	Low	Low
Qiu and Cui [19]	High	Moderate	High	Low	Low	Moderate	Moderate	Moderate	High	High	High	Low	Moderate
Xia et al. [20]	High	High	High	Moderate	Moderate	Moderate	High	High	High	High	High	High	High
Xiang et al. [21]	High	High	High	Moderate	Moderate	Moderate	High	High	High	High	High	Moderate	High
Pan et al. [22]	High	High	High	High	Moderate	Moderate	High	High	High	High	High	High	High
Xiang et al. [23]	High	High	High	Moderate	Moderate	High	High	High	High	High	High	Moderate	High
Perucho et al. [24]	Moderate	Moderate	Moderate	Low	Low	Moderate	Moderate	Moderate	High	High	Moderate	Moderate	Moderate
Perucho et al. [25]	High	High	High	Moderate	Moderate	Moderate	Moderate	High	High	High	Moderate	Moderate	High
Ferrer-Altabás et al. [26]	Moderate	Moderate	Moderate	Moderate	Moderate	Moderate	Moderate	Moderate	High	High	Moderate	High	Moderate
De Lestrange-Anginieur et al. [11]	High	High	High	High	Moderate	High	High	High	High	High	High	High	High
Trapp et al. [29]	High	High	High	Moderate	Moderate	Moderate	High	High	High	Moderate	High	Moderate	High
Trapp et al. [28]	High	High	High	Moderate	Moderate	Moderate	High	High	High	Moderate	High	Moderate	High
Lyu et al. [30]	Moderate	High	High	Moderate	Moderate	Moderate	High	High	High	High	High	High	High

Note on the quality assessment criteria: Each of the following 12 dimensions was rated as Low, Medium or High based on the specific criteria outlined below: (1) Sample Size / Model Parameter Rationality: *High* = parameters well-justified by theory or prior work; *Medium* = partially justified but not fully explained; *Low* = arbitrary or not explained; (2) Validity and Reliability of Measurement Tools: *High* = use of validated commercial equipment or standard methods; *Medium* = reasonable but not fully validated custom systems; *Low* = methods unspecified or clearly unreliable; (3) Appropriateness of Data Analysis Methods: *High* = use of mature and suitable methods; *Medium* = generally appropriate but not fully validated; *Low* = methods inappropriate or not specified; (4) Control of Confounding Factors: *High* = explicit control or discussion; *Medium* = partial control but not systematic; *Low* = not considered; (5) Research Process Transparency: *High* = code, data or detailed algorithms provided; *Medium* = methods described but code not public; *Low* = process descriptions vague; (6) Reproducibility of Results: *High* = methods thorough and fully reproducible; *Medium* = generally clear but lacking some details; *Low* = descriptions too brief for reproduction; (7) Comprehensiveness of Performance Evaluation: *High* = systematic multi-metric evaluation; *Medium* = key metrics evaluated but not comprehensively; *Low* = single or limited metrics used; (8) Comparative Analysis with Existing Techniques: *High* = systematic and quantitative comparison; *Medium* = comparison not systematic or only qualitative; *Low* = no comparison or only self-comparison; (9) Innovation and Originality: *High* = novel concept or method with clear originality; *Medium* = clear improvement with some novelty; *Low* = limited innovation, conventional application; (10) Technical Feasibility and Implementability: *High* = fully validated via experiments/prototypes; *Medium* = supported by simulation/theory but lacks physical validation; *Low* = remains conceptual, unvalidated; (11) Theoretical Rigour and Depth: *High* = rigorous model with complete derivation; *Medium* = theoretically reasonable but simplified; *Low* = weak theoretical support, vague; (12) Discussion of Limitations or Biases: *High* = explicit discussion of limitations; *Medium* = briefly mentioned but not explored; *Low* = not discussed.

### Freeform Surface Design

Freeform surface design became the predominant method for PALs by enabling the creation of complex optical surfaces tailored to the dynamic gaze demands of presbyopic patients. This approach partitioned the lens into micro-regions with dedicated optical axes, aiming to provide a more natural and continuous visual experience across varying distances. A key strategy was multi-optical-axis system design. Xiang et al. [15] integrated this approach into their freeform PALs design. They employed a small-pupil structure for the intermediate corridor and quadratic curves for the far and near vision zones. Their experimental prototypes demonstrated enhanced visual continuity, suggesting a potential reduction in visual jump and excessive head movement for presbyopes switching between viewing distances. Subsequently, Casanellas et al. [16] developed an optimisation model with spherical coordinates and interior point solvers, improving convexity control and computational stability. Galinier et al. [17] utilised a Fermat spiral design to create a spiral dioptre lens. By generating optical vortices, this method provides aperture-independent multifocality and an extended depth of field, ensuring consistent optical quality regardless of pupil constriction. Most recently, Yin et al. [18] complemented this with a customised method via localised precision optimisation, which enabled precise control in key visual zones and addressed directly the high demand of presbyopic patients for optical quality at intermediate and near distances, holding promise for reducing their visual fatigue. These works traced a clear progression in freeform surface design of PALs.

### Control of Surface Astigmatism

The control of surface astigmatism represented a pivotal challenge in PAL design, as it governed peripheral optical quality and wearer comfort directly. Uncontrolled astigmatism was a primary cause of blurred and distorted vision as well as the disorienting ‘swimming sensation’, which together posed a significant barrier to user adaptation and clinical acceptance. The studies included reflected an evolution in strategies to manage this fundamental aberration.

**Fig. 2:**
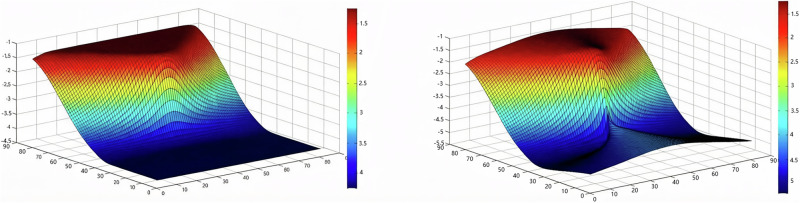
Comparison of dioptre distributions between a standard PAL and a PAL with directly added cylinder power (Reprinted with permission from [14]. © 2015 SPIE). The left figure illustrates the smooth dioptre distribution of a conventional PAL designed without astigmatism correction. The right figure shows the resulting dioptre distribution when cylinder power for astigmatism correction is directly added to the PAL’s diopter profile, highlighting the significant loss of surface smoothness and the emergence of optical discontinuities. In both plots, the horizontal axes (*X* and *Y*) represent the spatial coordinates across the lens surface (in mm), while the vertical axis (*Z*) and the colour scale denote the local dioptric power (in Dioptres, D). The transition from warm colours (red) to cool colours (blue) indicates a progression from lower to higher negative dioptric power, ranging approximately from −0.5 D to −5.5 D [14].

Early strategies employed direct superposition to integrate the astigmatic correction. Wei et al. [14] calculated the freeform surface height of astigmatism-free PALs and the cylindrical surface height for astigmatism correction separately before adding them. This approach prevented the disruption of the PAL’s smooth dioptric progression that would occur from directly adding cylinder power (Fig. 2), effectively creating a compound lens within a single optical surface. Similarly, Qiu et al. [19] introduced a hyperbolic tangential function to describe the desired power distribution on the lens, and used the Laplace equation to govern the dioptric distribution across the lens. This method leveraged the Laplace equation’s property of minimising surface gradient variations, thereby promoting a smoother power map and inherently constraining irregular surface astigmatism.

Later work shifted towards more sophisticated surface manipulation. Xia et al. [20] proposed a segmented freeform surface (SFS) method, which segmented the lens along the lines of curvature. Within each segment, the surface was locally reconfigured to equalise the two principal curvatures, thereby directly reducing the local astigmatism while largely preserving the intended mean power. Xiang et al. [21] found that the eighth-order polynomial outperformed linear, triangular, and direct assignment models in control of surface astigmatism and power stability for freeform PALs. Most recently, Pan et al. [22] proposed a differentiable optimisation method based on Non-Uniform Rational B-Spline (NURBS) surfaces, which minimised a nonlinear error function to push high astigmatic areas towards the lens edge.

Collectively, these studies documented a clear trajectory from simple corrective addition toward intelligent, algorithm-driven aberration management, aiming to optimise the astigmatism distribution across the lens surface for enhanced visual performance.

### Optical Performance Quantification

Objective and accurate evaluation of the optical performance of a PAL is crucial for translating optical designs into clinical benefits. Advanced techniques converted subjective complaints by the wearers into quantifiable parameters, thereby creating a precise feedback loop for designers. This shift moved lens optimisation from merely relying on vague patient descriptions to being driven by solid objective data, which facilitated the more efficient development of PALs that satisfied presbyopic patients.

Xiang et al. [23] introduced a multi-optical-axis measurement technique using a Hartmann–Shack wavefront sensor to quantify spherical and astigmatic power and represented the results visually as contour maps. Their results showed a measurable reduction in surface astigmatism in the tested design, particularly in the nasal region of the near vision zone, thus providing a quantifiable target for control of surface astigmatism. Complementing this, Gabor holography was applied extensively for quality inspection. Perucho et al. [24, 25] developed wavefront holoscopy (w-holoscope) for magnified visualisation of the permanent engraved marks (PEMs), offering an economical solution for quality inspection and assisting practitioners in identifying lens parameters in challenging clinical scenarios. Ferrer-Altabás [26] further advanced this with ‘Shadowfocimetry,’ which integrated Gabor holographic principles into a manual focimeter for accurate localisation and quantification of the angular deviation. Additionally, De Lestrange-Anginieur et al. [11] developed novel optical metrics sensitive to the surface power changes in a PAL, validating their effectiveness in assessing optical performance quantitatively, particularly in regard to astigmatism.

Although objective optical metrics drove significant advancements in PAL design, these improvements did not always correspond directly to wearer satisfaction or adaptation, which is the final criterion for evaluating the quality of PAL design. For instance, De Lestrange-Anginieur et al. [11] demonstrated that conventional high-contrast visual acuity measures correlated only weakly with users’ actual preferences, underscoring the limitations of relying solely on objective parameters to predict subjective experience. Some PAL designs that performed exceptionally well in optical assessments may still cause adaptation difficulties for certain users. This disparity indicated that, in addition to optical performance, the physiological adaptation mechanisms played a crucial role in determining the subjective experience of the wearer [27]. While objective metrics could quantify optical performance precisely, they often failed to capture the complex interplay of visual comfort, adaptation and neural processing that defined a wearer’s real-world experience. However, few studies linked the wearer’s preference data directly with the different designs of the PAL, leaving a recognised gap in linking these optical measurements to their validation against subjective outcomes.

### Emerging Lens Concepts

The advent of new technologies offers broad possibilities for developing novel lenses with higher diffraction efficiency, lower chromatic aberration, wider viewing angles or extended depth of focus, foreshadowing a diversified and high-performance future for PALs. These emerging concepts addressed specific limitations highly relevant to presbyopic patients. For instance, the lightweight and thin profile of holographic PALs [28, 29] potentially overcame the cosmetic and comfort concerns associated with the centre thickness and weight of traditional PALs, especially for patients requiring high prescriptions. Concurrently, extended depth-of-focus (EDoF) lens designs [30] targeted a more seamless transition from far to near, catering to the modern presbyope’s need for ‘seamless vision’ in dynamic daily tasks, such as shifting gaze between the road and the dashboard while driving. Although current EDoF designs are sensitive to pupil size and decentration, this highlights the need for future optimisation tailored to the specific physiological conditions of different presbyopic subpopulations.

## Discussion

This systematic review synthesised a decade of advancements in PALs design, charting a clear trajectory from incremental improvements in optical performance toward the ultimate goal of personalised, human-centric solutions. The collective findings reveal a field in transition, where the core design philosophy evolved from merely correcting aberrations to intelligently managing them, a shift enabled by sophisticated freeform surfaces and algorithmic optimisation. Concurrently, the field was augmented by innovative optical performance metrics that enabled more objective assessment of wearer experience, despite the persistent gap between optical perfection and subjective satisfaction.

The fundamental challenge in PAL design lies in the inescapable trade-off between achieving a seamless progression of optical power and minimising peripheral aberrations. The maturation of freeform surface technology represented the principal response to this challenge, enabling the creation of complex, high-order optical surfaces. Early strategies focused on the control of surface astigmatism through surface superposition or analytical functions [14, 19]. The field evolved towards a more sophisticated paradigm of intelligent aberration management, as exemplified by the use of high-order polynomials [21] or NURBS surfaces [22]. These approaches did not seek to eliminate aberrations entirely, but instead systematically drove them away from critical visual zones towards the lens periphery. This marked a significant philosophical shift from mere correction to optimised distribution, and moved beyond experiential fitting towards a physics-informed, algorithmic design process.

The optimisation of complex freeform designs necessitated equally advanced verification methods. Historically, the evaluation of PALs relied heavily on subjective wearer feedback, which was qualitative and variable. Recent innovations in objective optical metrology established a crucial feedback loop for design refinement. Techniques such as Hartmann–Shack wavefront sensing [23] provided direct, quantitative maps of optical power and astigmatism, while Gabor holography-based methods like wavefront holoscopy [24, 25] and Shadowfocimetry [26] offered robust, economical solutions for quality control and clinical measurement. These tools translated elusive subjective experiences like ‘blur’ and ‘swimming’ into quantifiable parameters and enabled data-driven design iterations and a more direct connection between optical engineering and clinical performance.

However, a certain degree of disconnect persisted between clinically used objective optometric measurements and the subjective visual experiences of PALs wearers. This gap was evident on three fronts. First, conventional laboratory and clinical metrics often failed to predict real-world preference. Although researchers had proposed advanced methods and indicators that could better quantify the subjective experiences of PAL wearers into measurable objective metrics, few studies quantitatively correlated these objective improvements with real-world wearer satisfaction, adaptation time or reduction in symptoms. This highlighted a critical disconnect that the designs became optically superior, but the evidence linking this to a better user experience remained largely indirect.

Second and perhaps more critically, the clinical pipeline itself was compromised by a lack of standardisation. Discrepancies between devices in measuring critical parameters like fitting height and pantoscopic tilt directly caused inaccuracies in PAL’s optical zone positioning and fitting centre placement [31, 32]. This lack of measurement consistency and reliability not only compromised the comparability and reproducibility of research data but also negated the potential benefits that sophisticated optical designs, no matter how well-intentioned, could otherwise have delivered to wearers. Future efforts should promote standardised measurement protocols, potentially referencing established frameworks such as International Standards Organization (ISO) 21987 for ophthalmic lenses, to ensure consistency across clinical and research settings.

Finally, a fundamental limitation underlying the subjective-objective disconnect was the predominant focus on the physical characterisation of optical aberrations [11], which largely overlooked the critical role played by the wearer’s own physiological mechanisms in processing and adapting to distorted visual information [27, 33]. This neglect meant that complex dynamic factors, such as visual compensation and neural adaptation, were insufficiently considered in the design process. For instance, the ‘swimming sensation’ and motion discomfort reported by wearers were linked to gaze-contingent distortions of optic flow that predictably altered the perception of self-motion direction [34]. While recent studies began to investigate contributing factors like eye movements [35] and vergence flexibility [36], the precise neural basis and individual variability in neural plasticity thresholds were still unquantified. Although progress was made in quantifying these perceptual distortions objectively, research into the underlying mechanisms was still in its early stages [37]. This gap limited the ability to predict a user’s adaptation potential and ultimately impeded the transition from simple optical compensation to achieving true neuro-optical synergy in PAL design.

In the future, PALs will shift from a static, parameter-driven product to a dynamic, behaviour-aware visual wearable [38]. This transformation will be powered by the integration of wearable eye-tracking and computer vision technologies [39, 40] and human factors frameworks, enabling continuous capture of users’ real-world visual behaviour and posture. It will provide holistic metrics to guide lens optimisation and enable truly personalised optical solutions [41–43]. Through cloud platforms, optometric data, 3D facial scans, frame parameters and user preferences can be integrated to form a collaborative design ecosystem. Advances in artificial-intelligence (AI)-driven design, new optical materials and 3D printing will accelerate this shift [44, 45]. The integration of virtual reality (VR)/augmented reality (AR) for immersive testing will also be crucial [46]. This progress highlights the growing importance of multi-disciplinary integration across optics, computer science, materials science and visual physiology [47]. The ultimate goal is to transform PALs from a standardised product into a truly personalised visual wearable device.

## Conclusion

PALs serve as essential optical devices for correcting presbyopia, with their design and evaluation technologies evolving continuously. This systematic review synthesises current advancements in PALs regarding freeform surface design, control of surface astigmatism, advanced optical evaluation methods and novel optical elements. Despite existing challenges, ongoing technological innovations and deeper interdisciplinary integration are poised to enable future PALs to deliver increasingly personalised and comfortable visual experiences, thereby significantly enhancing the quality of life for presbyopic patients worldwide.

## Supplementary information

Supplementary Materials

## Data Availability

No datasets were generated or analysed during the current study.

## References

[1] Wolffsohn JS, Davies LN. Presbyopia: effectiveness of correction strategies. Prog Retin Eye Res. 2019;68:124–43. 10.1016/j.preteyeres.2018.09.004. 30244049 10.1016/j.preteyeres.2018.09.004

[2] Holden BA, Fricke TR, Ho SM, Wong R, Schlenther G, Cronje S, et al. Global vision impairment due to uncorrected presbyopia. Arch Ophthalmol. 2008;126:1731–9. 10.1001/archopht.126.12.1731. 19064856 10.1001/archopht.126.12.1731

[3] Bourne RRA, Flaxman SR, Braithwaite T, Cicinelli MV, Das A, Jonas JB, et al. Magnitude, temporal trends, and projections of the global prevalence of blindness and distance and near vision impairment: a systematic review and meta-analysis. Lancet Glob Health. 2017;5:e888–e97. 10.1016/S2214-109X(17)30293-0. 28779882 10.1016/S2214-109X(17)30293-0

[4] Berdahl J, Bala C, Dhariwal M, Lemp-Hull J, Thakker D, Jawla S. Patient and economic burden of presbyopia: a systematic literature review. Clin Ophthalmol. 2020;14:3439–50. 10.2147/OPTH.S269597. 33116396 10.2147/OPTH.S269597 PMC7588278

[5] Goertz AD, Stewart WC, Burns WR, Stewart JA, Nelson LA. Review of the impact of presbyopia on quality of life in the developing and developed world. Acta Ophthalmol. 2014;92:497–500. 10.1111/aos.12308. 24910300 10.1111/aos.12308

[6] Katz JA, Karpecki PM, Dorca A, Chiva-Razavi S, Floyd H, Barnes E, et al. Presbyopia - a review of current treatment options and emerging therapies. Clin Ophthalmol. 2021;15:2167–78. 10.2147/OPTH.S259011. 34079215 10.2147/OPTH.S259011 PMC8163965

[7] Charman WN. Developments in the correction of presbyopia I: spectacle and contact lenses. Ophthalmic Physiol Opt. 2014;34:8–29. 10.1111/opo.12091. 24205890 10.1111/opo.12091

[8] Jamali A, Bryant D, Bhowmick AK, Bos PJ. Large area liquid crystal lenses for correction of presbyopia. Opt Express. 2020;28:33982–93. 10.1364/OE.408770. 33182876 10.1364/OE.408770

[9] Hoster M. Get to know your PALs: a step up from standard bifocals and trifocals, progressive addition lenses are an attractive option for many presbyopes. Here is a look at some of the latest designs. Rev Optom. Rev Optom. 2010;147:39. https://www.reviewofoptometry.com/article/get-to-know-your-pals.

[10] Ahmad Najmee NA, Buari NH, Mujari R, Rahman MI. Quality of vision of presbyopic via progressive additional lens (PALs). Asian J Qual Life. 2018;3:150–9. 10.21834/ajqol.v3i13.171.

[11] De Lestrange-Anginieur E, Kee CS. Optical performance of progressive addition lenses (PALs) with astigmatic prescription. Sci Rep. 2021;11:2984. 10.1038/s41598-021-82697-0. 33542417 10.1038/s41598-021-82697-0 PMC7862262

[12] Forkel J, Reiniger JL, Muschielok A, Welk A, Seidemann A, Baumbach P. Personalized progressive addition lenses: correlation between performance and design. Optom Vis Sci. 2017;94:208–18. 10.1097/OPX.0000000000001016. 27918396 10.1097/OPX.0000000000001016

[13] Barbero S, Portilla J. The relationship between dioptric power and magnification in progressive addition lenses. Ophthalmic Physiol Opt. 2016;36:421–7. 10.1111/opo.12301. 27146008 10.1111/opo.12301

[14] Wei Y, Xiang H, Zhu T, Chen J. A design of PAL with astigmatism. In: Wang Y, Tan X, Tatsuno K (Eds), Proceedings of SPIE: Vol. 9618. International Conference on Optical Instruments and Technology: Optical Systems and Modern Optoelectronic Instruments. Bellingham, WA, USA: SPIE; 2015. p. 961819. 10.1117/12.2196275.

[15] Xiang H, Chen J, Zhu T, Wei Y, Fu D Theoretical and experimental investigation of design for multioptical-axis freeform progressive addition lenses. Opt Eng. 2015;54 10.1117/1.OE.54.11.115110.

[16] Casanellas G, Castro J. Using interior point solvers for optimizing progressive lens models with spherical coordinates. Optim Eng. 2020;21:1389–421. 10.1007/s11081-019-09480-z.

[17] Galinier L, Renaud-Goud P, Brusau J, Kergadallan L, Augereau J, Simon B. Spiral diopter: freeform lenses with enhanced multifocal behavior. Optica. 2024;11:238–44. 10.1364/optica.507066.

[18] Yin Z, Wang L, Zhang X, Zeng X, Liu Y, Hu J. Design of customized freeform progressive addition lens based on localized precision optimization. Opt Express. 2025;33:23036–52. 10.1364/OE.564129. 40515277 10.1364/OE.564129

[19] Qiu G, Cui X. Hyperbolic tangential function-based progressive addition lens design. Appl Opt. 2015;54:10404–8. 10.1364/ao.54.010404. 26836863 10.1364/AO.54.010404

[20] Xia R, Fu Y, Ma K, Chen S, Pan J, Zhou C, et al. Surface astigmatism correction using segmented freeform surfaces for a progressive addition lens. Opt Express. 2022;30:43384–97. 10.1364/OE.476678. 36523037 10.1364/OE.476678

[21] Xiang HZ, Zhang X, Gao JD, Wang P, Ding QH, Li HT, et al. Comparison of different power distributions for designing freeform progressive addition lenses based on a minimization error function model. Opt Eng. 2022;61 10.1117/1.Oe.61.3.035106.

[22] Pan X, Tang H, Feng Z, Xiang H. Differentiable design of progressive-addition lens using NURBS surface. Opt Express. 2025;33:10485–97. 10.1364/oe.551518. 40798697 10.1364/OE.551518

[23] Xiang HZ, Guo H, Fu DX, Zheng G, Zhuang SL, Chen JB, et al. Multi-optical-axis measurement of freeform progressive addition lenses using a Hartmann-Shack wavefront sensor. Opt Lasers Eng. 2018;104:259–65. 10.1016/j.optlaseng.2017.11.003.

[24] Perucho B, Picazo-Bueno J A, Ferreira C, Micó V, editors. Manual wavefront holoscopy for inspection and visualization of engraved marks in progressive addition lenses. In: Conference on optical methods for inspection, characterization, and imaging of biomaterials II. Munich, Germany; SPIE, Bellingham, WA, USA. 2015 10.1117/12.2184787.

[25] Perucho B, Picazo-Bueno JA, Micó V. A novel marking reader for progressive addition lenses based on gabor holography. Optom Vis Sci. 2016;93:534–42. 10.1097/opx.0000000000000818. 26855243 10.1097/OPX.0000000000000818

[26] Ferrer-Altabás S, Picazo-Bueno JA, Granero-Montagud L, Micó V. Shadowfocimetry: adapting the holographic principle to a manual focimeter for visualization/marking of permanent engravings in progressive addition lenses. Opt Lett. 2022;47:2298–301. 10.1364/ol.454962. 35486785 10.1364/OL.454962

[27] Habtegiorgis SW, Jarvers C, Rifai K, Neumann H, Wahl S. The role of bottom-up and top-down cortical interactions in adaptation to natural scene statistics. Front Neural Circuits. 2019;13:9 10.3389/fncir.2019.00009. 30814934 10.3389/fncir.2019.00009 PMC6381060

[28] Trapp JM, Decker M, Petschulat J, Pertsch T, Jabbour TG. Design of a 2 diopter holographic progressive lens. Opt Express. 2018;26:32866–77. 10.1364/oe.26.032866. 30645447 10.1364/OE.26.032866

[29] Trapp JM, Decker M, Petschulat J, Pertsch T, Jabbour TG, editors. Holographic progressive lenses. In: 19th conference on current developments in lens design and optical engineering. San Diego, California; (SPIE, Bellingham, WA, USA. 2018).

[30] Lyu J, Bang SP, Yoon G. Refractive extended depth-of-focus lens design based on periodic power profile for presbyopia correction. Ophthalmic Physiol Opt. 2024;44:301–10. 10.1111/opo.13253. 37984831 10.1111/opo.13253 PMC10925839

[31] Garcia-Espinilla O, Gallegos-Cocho I, Sanchez I, Cañadas P, Martin R. Comparison of physiognomy and frame angle parameters using different devices to prescribe progressive addition lenses. Clin Exp Optom. 2022;105:420–7. 10.1080/08164622.2021.1914511. 33971792 10.1080/08164622.2021.1914511

[32] Garcia-Espinilla O, Gallegos-Cocho I, Sanchez I, Cañadas P, Martin R. Interdevice agreement in the measurement of physiognomy parameters and frame angles to prescribe progressive addition lenses. Clin Exp Optom. 2023;106:69–74. 10.1080/08164622.2021.2006042. 35000565 10.1080/08164622.2021.2006042

[33] Garcia Garcia M, Rifai K, Wahl S, Watson T. Adaptation to geometrically skewed moving images: An asymmetrical effect on the double-drift illusion. Vis Res. 2021;179:75–84. 10.1016/j.visres.2020.11.008. 33310640 10.1016/j.visres.2020.11.008

[34] Sauer Y, Scherff M, Lappe M, Rifai K, Stein N, Wahl S. Self-motion illusions from distorted optic flow in multifocal glasses. iScience. 2022;25:103567. 10.1016/j.isci.2021.103567. 34988405 10.1016/j.isci.2021.103567 PMC8693457

[35] Concepcion-Grande P, González A, Chamorro E, Cleva JM, Alonso J, Gómez-Pedrero JA. Eye movements as a predictor of preference for progressive power lenses. J Eye Mov Res. 2022;15 10.16910/jemr.15.2.6. 10.16910/jemr.15.2.6 PMC9669734 36405238

[36] Alvarez TL, Kim EH, Granger-Donetti B. Adaptation to progressive additive lenses: potential factors to consider. Sci Rep. 2017;7:2529. 10.1038/s41598-017-02851-5. 28566706 10.1038/s41598-017-02851-5 PMC5451391

[37] Sauer Y, Künstle DE, Wichmann FA, Wahl S. An objective measurement approach to quantify the perceived distortions of spectacle lenses. Sci Rep. 2024;14 10.1038/s41598-024-54368-3. 10.1038/s41598-024-54368-3 PMC10874444 38368485

[38] Tang Y, Wu Q, Chen X, Zhang H. A personalized design for progressive addition lenses. Opt Express. 2017;25:28100 10.1364/OE.25.028100.

[39] Lin S-F, Yuan S-M, Liu X, Zhang X, Mehmood I. Eye tracking based control system for natural human-computer interaction. Comput Intell Neurosci. 2017;2017:1–9. 10.1155/2017/5739301. 10.1155/2017/5739301 PMC5748315 29403528

[40] Novák JŠ, Masner J, Benda P, Šimek P, Merunka V. Eye tracking, usability, and user experience: a systematic review. Int J Hum-Comput Interact. 2024;40:4484–500. 10.1080/10447318.2023.2221600.

[41] Kolbe O, Degle S. Presbyopic personal computer work: a comparison of progressive addition lenses for general purpose and personal computer work. Optom Vis Sci. 2018;95:1046–53. 10.1097/opx.0000000000001295. 30339644 10.1097/OPX.0000000000001295

[42] Kolbe O, Bitterlich K, Lahne J, Degle S, Anders C. Surface electromyography of the trapezius and sternocleidomastoid during computer work with presbyopic corrections. Optom Vis Sci. 2022;99:496–504. 10.1097/opx.0000000000001899. 35412478 10.1097/OPX.0000000000001899

[43] Kolbe O, Becker P, Degle S, Anders C. Trapezius activity during personal computer work with progressive addition lenses for general purpose and for computer work in neophytes. Ophthalmic Physiol Opt. 2023;43:1391–405. 10.1111/opo.13196. 37417310 10.1111/opo.13196

[44] Fan D, Smith CS, Unnithan RR, Kim S. 3D printed diffractive optical elements for rapid prototyping. Micro Nano Eng. 2024;24:100270. 10.1016/j.mne.2024.100270.

[45] You R, Hong Z, Chen J, Zhang Z, Wang Y, Sun Y, et al. Extremely compact 3D printed glass ternary diffractive optical element for holographic images. Adv Opt Mater. 2025;13:n/a 10.1002/adom.202501074. 10.1002/adom.202501074 PMC12369660 40852131

[46] Yin K, Hsiang E-L, Zou J, Li Y, Yang Z, Yang Q, et al. Advanced liquid crystal devices for augmented reality and virtual reality displays: principles and applications. Light Sci Appl. 2022;11:161–22. 10.1038/s41377-022-00851-3. 35637183 10.1038/s41377-022-00851-3 PMC9151772

[47] Mahmoud CF, Sørensen JT. Artificial intelligence in personalized learning with a focus on current developments and future prospects. Res Adv Educ. 2024;3:25–31. 10.56397/rae.2024.08.04.

