# Supplementary material for: Design of Progressive Addition Lens for Presbyopia: A Systematic Review
Source: Ophthalmic Physiol Opt. 2026 Apr 22;46(2):395–403. doi: 10.1007/s44402-026-00056-w (PMC13369678; doi:10.1007/s44402-026-00056-w)
Supplement: Supplementary file 1 — Supplementary Materials [file 44402_2026_56_MOESM1_ESM.doc]

**Supplement 1. Details of the Search Strategy**

| **Search Strategy for each database** | |
| --- | --- |
| PubMed | ((((presbyopia[MeSH Major Topic]) OR (presbyopia[Title/Abstract])) OR (presbyopic[Title/Abstract])) OR (aging eye[Title/Abstract])) AND ((((((progressive addition lens[Title/Abstract]) OR (progressive lens[Title/Abstract])) OR (PALs[Title/Abstract])) OR (multifocal glasses[Title/Abstract])) OR (varifocal lens[Title/Abstract])) OR (lens design[Title/Abstract])) |
| EMBASE | ('presbyopia'/exp OR presbyopia:ti,ab OR presbyopic:ti,ab OR 'aging eye':ti,ab) AND ('progressive addition lens':ti,ab OR 'progressive lens':ti,ab OR 'pals':ti,ab OR 'multifocal glasses':ti,ab OR 'varifocal lens':ti,ab OR 'freeform lens':ti,ab OR 'lens design':ti,ab) |
| Cochrane Library | (MeSH descriptor: [Presbyopia] explode all trees OR (presbyopia):ti,ab,kw OR (presbyopic):ti,ab,kw OR ("aging eye"):ti,ab,kw) AND (("progressive addition lens"):ti,ab,kw OR ("progressive-addition lenses"):ti,ab,kw OR ("Progressive Lens"):ti,ab,kw OR ("Multifocal Glasses"):ti,ab,kw OR ("Varifocal Lens"):ti,ab,kw OR ("Freeform Lens"):ti,ab,kw OR ("Lens Design"):ti,ab,kw) |
| Web of science | TS=(Presbyopia OR Presbyopic OR "Aging Eye") AND TS=("Progressive Addition Lens" OR "Progressive Lens" OR PALs OR "Multifocal Glasses" OR "Varifocal Lens" OR "Freeform Lens" OR "Lens Design") |
| Scopus | ( ( TITLE-ABS-KEY ( "aging eye" ) OR TITLE-ABS-KEY ( presbyopia ) OR TITLE-ABS-KEY ( presbyopic ) ) ) AND ( ( TITLE-ABS-KEY ( "Progressive Addition Lens" ) OR TITLE-ABS-KEY ( "Progressive Lens" ) OR TITLE-ABS-KEY ( PALs ) OR TITLE-ABS-KEY ( "Multifocal Glasses" ) OR TITLE-ABS-KEY ( "Varifocal Lens" ) OR TITLE-ABS-KEY ( "Freeform Lens" ) OR TITLE-ABS-KEY ( "Lens Design" ) ) ) |
| SPIE Digital Library | (TITLE:(presbyopia) OR TITLE:(presbyopic) OR TITLE:(“aging eye”) OR ABSTRACT:(presbyopia) OR ABSTRACT:(presbyopic) OR ABSTRACT:(“aging eye”)) AND (TITLE:("progressive addition lens") OR TITLE:("progressive lens") OR TITLE:("PALs”) OR TITLE:("multifocal glasses") OR TITLE:("varifocal lens") OR TITLE:("freeform lens") OR TITLE:("lens design") OR ABSTRACT:("progressive addition lens") OR ABSTRACT:("progressive lens") OR ABSTRACT:("PALs") OR ABSTRACT:("multifocal glasses") OR ABSTRACT:("varifocal lens") OR ABSTRACT:("freeform lens") OR ABSTRACT:("lens design")) |

**Supplement 2. Details of the Assessment Criteria**

1. **Sample Size / Model Parameter Rationaly:**

High: Parameters are well-justified by theory or prior studies.

Medium: Parameters are partially justified but not fully explained.

Low: Parameters are arbitrary or not explained.

1. **Validity and Reliability of Measurement Tools:**

High: Use of validated commercial equipment or standard methods.

Medium: Use of reasonable but not fully validated custom systems.

Low: Methods are unspecified or clearly unreliable.

1. **Appropriateness of Data Analysis Methods:**

High: Use of mature and suitable statistical or numerical methods.

Medium: Methods are generally appropriate but not fully validated.

Low: Methods are inappropriate or not specified.

1. **Control of Confounding Factors:**

High: Explicit control or discussion of confounding factors.

Medium: Partial control but not systematically addressed.

Low: No consideration of confounding factors.

1. **Research Process Transparency:**

High: Code, data, or detailed algorithms are provided.

Medium: Methods are described but code is not publicly available.

Low: Process descriptions are vague.

1. **Reproducibility of Results:**

High: Methods are thoroughly described and fully reproducible.

Medium: Methods are generally clear but lack some details.

Low: Descriptions are too brief or lack critical information for reproduction.

1. **Comprehensiveness of Performance Evaluation:**

High: Systematic evaluation using multiple dimensions and metrics.

Medium: Key metrics are evaluated but not comprehensively.

Low: Evaluation relies on single or limited metrics.

1. **Comparative Analysis with Existing Techniques:**

High: Systematic and quantitative comparison with existing techniques.

Medium: Comparison is made but not systematic or only qualitative.

**Low: No comparison with existing techniques, or only self-comparison.**

1. **Innovation and Originality:**

High: Introduces a novel concept or method with clear originality.

Medium: Clear improvement over existing techniques with some novelty.

Low: Limited innovation, primarily conventional application or minor modification.

1. **Technical Feasibility and Implementability:**

High: Fully validated through experiments or prototypes with high implementability.

Medium: Supported by simulation or theory but lacks physical validation.

Low: Remains conceptual without feasibility validation.

1. **Theoretical Rigor and Depth:**

High: Rigorous theoretical model with complete derivation and sufficient depth.

Medium: Theoretically reasonable but somewhat simplified or moderate in depth.

Low: Weak theoretical support with vague descriptions.

1. **Discussion of Limitations or Biases:**

High: Explicit discussion of study limitations.

Medium: Briefly mentioned but not explored in depth.

Low: No discussion of limitations.
